# Dengue epidemic in Belém, Pará, Brazil, 1996-97.

**DOI:** 10.3201/eid0603.000311

**Published:** 2000

**Authors:** A P Trravassos da Rosa, P F Vasconcelos, E S Travassos Da Rosa, S G Rodrigues, B Mondet, A C Cruz, M R Sousa, J F Travassos Da Rosa

**Affiliations:** Instituto Evandro Chagas, Belém, Pará, Brazil. pedrovasconcelos@iec.pa.gov.br

## Abstract

We describe clinical and epidemiologic findings during the first epidemic of dengue fever in Belém, Pará State, Brazil, in 1996-97. Of 40,237 serum samples, 17,440 (43%) were positive for dengue by virus isolation or serologic testing. No hemorrhagic cases or deaths were reported. Mycobacterium tuberculosis


### Dengue Epidemic in Belém, Pará, Brazil, 1996–97

**Amélia P.A. Travassos da Rosa, Pedro F.C. Vasconcelos, Elizabeth S. Travassos da Rosa, Sueli G. Rodrigues, Bernard Mondet, Ana C.R. Cruz, Maria R. Sousa, and Jorge F.S. Travassos da Rosa**
Instituto Evandro Chagas, Belém, Pará, Brazil

We describe clinical and epidemiologic findings during the first epidemic of dengue fever in Belém, Pará State, Brazil, in 1996-97. Of 40,237 serum samples, 17,440 (43%) were positive for dengue by virus isolation or serologic testing. No hemorrhagic cases or deaths were reported.

Dengue fever (DF) and dengue hemorrhagic fever (DHF) are caused by infection with one of the four serotypes of dengue virus (DEN-1, DEN-2, DEN-3, and DEN-4), transmitted by ***Aedes aegypti*** mosquitoes. In Brazil, DF epidemics reported in the 1980s and 1990s involved more than a million cases. However, only 671 DHF cases were diagnosed, with 26 deaths (1).

***Ae. aegypti*** was reintroduced in Pará State in 1992, and the first dengue cases were reported in 1995 in the southeast region (Redenção and Rondon do Pará). In October 1996, eight cases of febrile denguelike illness were reported in Belém (population 1,300,000), a city in the Brazilian Amazon region at the confluence of the Amazon River and the Atlantic Ocean. In early November, DEN-1 virus was isolated and identified (2-4). DEN-2 virus was identified in October 1997, and since then, both serotypes have been responsible for illness in Belém. This was the first time dengue virus transmission occurred in Belém during the last 70 years and the third time the disease occurred in the Brazilian Amazon region. Previous outbreaks had been reported in 1981-82 in Boa Vista, Roraima (5), and in 1991 in Araguaina, Tocantins State (6).

We describe cases of denguelike illness diagnosed at Instituto Evandro Chagas. A case of dengue was defined as illness with the following symptoms: acute onset of high fever, headache, myalgia, arthralgia, dizziness, and other symptoms and signs suggestive of denguelike illness in a patient with a positive IgM by IgM-capture enzyme-linked immunosorbent assay (MAC ELISA), virus isolation or serologic conversion in paired serum samples, and an increase of at least fourfold in titer in the convalescent-phase serum sample (7-9).

Address for correspondence: Pedro Fernando da Costa Vasconcelos, Instituto Evandro Chagas, Av. Almirante Barroso 492, 66090-000, Belém, Pará, Brazil; fax: 55-91-226-1284; e-mail: pedrovasconcelos@iec.pa.gov.br.

### The Study

From January to December 1997, 40,237 serum samples were drawn from febrile patients in Belém, 20,038 (49.8%) of whom were male. The patients were all residents of the municipalities of Belém (31,506 samples) and Ananindeua (8,731 samples). Most were ambulatory patients seen in the Arbovirus Department, Evandro Chagas Institute; some patients were referred by public health centers, private physicians, and clinics. At the Institute, all patients were examined clinically and had blood samples drawn. A questionnaire was administered that included information about clinical symptoms and signs and demographic data. Patients who had been ill <10 days and whose serologic tests were negative for dengue were requested to provide a second blood sample 7 to 14 days later. For patients who had at least one hemorrhage and either dehydration or hemoconcentration, a leukogram, platelet count, and hematocrit were performed.

Serum samples were tested for dengue antibodies by MAC ELISA (4) and hemagglutination-inhibition test (2). The antigens for both tests were prepared by using infected mouse brain extracted by the sucrose acetone method. The criteria used for establishing primary and secondary infection were those recommended by the World Health Organization (7,8).

To isolate dengue virus, 0.1-ml aliquots of whole blood from patients with clinical symptoms lasting <5 days and negative serologic results were injected into cultures of C6/36 cells (10). The cultures were visually examined daily, and cells were tested on days 7 and 14 by immunofluorescence (3).

Isolated virus strains were initially screened by direct immunofluorescence against a flavivirus standard hyperimmune fluid prepared at the Institute. Strains that reacted were identified to serotype by using an indirect immunofluorescence test with monoclonal antibodies against the four dengue viruses provided by the Centers for Disease Control and Prevention.

The epidemic distribution was accompanied early in 1997 by a seasonal increase in rainfall typical of the Brazilian Amazon. However, the highest dengue positivity rates were reported in the dry months of September to December. Coincidentally, DEN-2 virus was isolated in October. At first, cases were reported only in Belém and Ananindeua, but in December 1997, at least 15 other municipalities reported transmission cycles involving either DEN-1 or DEN-2 or both.

Of all sera collected, 17,525 (43.5%) were positive by serologic testing or DEN virus isolation: 13,805 (78.8%) from Belém and 3,720 (21.2%) cases from Ananindeua (Figure). Both primary and secondary serologic responses were found. Among the positive samples, 9,469 (54.25%) were among female and 7,971 (45.75%) among male patients ($p < 0.0001$); 45.95% of the patients were 25 to 44 years of age (Table 1). Paired serum samples were obtained from 3,558 patients, 2,997 (84.2%) of whom had serologic conversions.

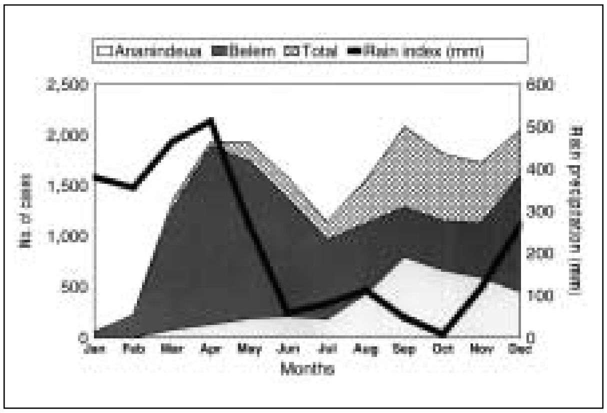

Figure. Monthly distribution of rain precipitation (line) and dengue fever cases as seen at Instituto Evandro Chagas, Pará, Brazil. Source for rain precipitation, Instituto Nacional de Meteorologia, Brazil.

No DHF cases were reported, in spite of the fact that since October 1997 DEN-2 had been isolated from 24 patients with previous DEN-1 infection (Table 2). Clinical symptoms were more severe in older children and adults than in young children, but no differences were observed between the clinical symptoms of patients with DEN-1 and DEN-2 infection.

Table 1. Distribution of patients with serologic tests positive for dengue

| | Male | | | Female | | | Total | |
|---|---|---|---|---|---|---|---|---|
| Age | No. | Pos | % | No. | Pos | % | Pos | % |
| <4 | 186 | 60 | 0.76 | 325 | 76 | 0.8 | 136 | 0.78 |
| 5–9 | 308 | 89 | 1.12 | 425 | 186 | 1.96 | 275 | 1.58 |
| 10–14 | 1,033 | 444 | 5.56 | 816 | 356 | 3.76 | 800 | 4.59 |
| 15–24 | 4,080 | 1,596 | 20 | 3,765 | 1,643 | 17.35 | 3,239 | 18.56 |
| 25–34 | 5,083 | 2,184 | 27.37 | 4,375 | 2,127 | 22.47 | 4,311 | 24.7 |
| 35–44 | 3,919 | 1,684 | 21.1 | 4,638 | 2,023 | 21.37 | 3,707 | 21.25 |
| 45–54 | 3,319 | 1,016 | 12.73 | 3,572 | 1,558 | 16.46 | 2,574 | 14.75 |
| >55 | 2,110 | 898 | 11.36 | 2,283 | 1,500 | 15.83 | 2,398 | 13.79 |
| Total | 20,038 | 7,971 | 100 | 20,199 | 9,469 | 100 | 17,440 | 100 |

Table 2. Strains of dengue virus isolated from patients from Belém and Ananindeua, Instituto Evandro Chagas, October 1996 to August 1998

| | DEN-1 | DEN-2 | Total |
|---|---|---|---|
| 1996 | 31 | - | 31 |
| 1997 | 751 | 48 | 799 |
| 1998[a] | 58 | 60 | 118 |
| Total | 840 | 108 | 948 |

[a]Through August.

### Conclusions

During 1953 and 1954, Causey and Theiler (11) used seroneutralization in mice to survey several municipalities of the Amazon region; they found 9.8% and 2.2% of serum samples positive for dengue virus serotypes DEN-1 and DEN-2, respectively, among residents > 50 years of age. These results suggest that dengue viruses were circulating in Belém and other municipalities of the Amazon Valley early in the 20th century.

The dengue epidemic in Belém was unusual in that a “lag phase” (12) lasted for at least 4 months before extensive transmission began. In spite of laboratory diagnosis of cases from the beginning of the outbreak and the reporting of these results to health authorities, control measures were unsuccessful. Consequently, explosive transmission began in March 1997 and is still occurring (1998-99).

In the last 4 years, the Brazilian Ministry of Health (13) has reported an increase in dengue cases from approximately 56,000 in 1994 to >530,000 in 1998. High indexes of ***Ae. aegypti*** infestation exist in all important urban centers of Brazil, reflecting a poor health education program. The fact that more cases occurred in female than in male patients ($p < 0.0001$) suggests that women are at increased risk for dengue infection because of high peridomestic exposure.

Dengue transmission has been reported in 22 of the 27 Brazilian states, and the mosquito vector is present in all states; therefore, the situation in Brazil may be rapidly approaching hyperendemicity, with the cocirculation of two serotypes (DEN-1 since 1986 and DEN-2 since 1990). The risk for DHF will increase if a new serotype (DEN-3 or DEN-4) is introduced. Endemicity is the most constant factor associated with the evolution of epidemic DHF in a geographic area (14). Although few cases have been reported until recently, DHF may become an important cause of hospitalization and death in the Americas, including Brazil.

### Acknowledgments

The authors thank Marcio R.T. Nunes, Gisele C.A. Barra, Denise I. Cerqueira, Nelma Mesquita, Andes K. Mahagama, Eliana Pinto, and Mioni T.F. Magalhães, and the Instituto Nacional de Meteorologia (Ministry of Agriculture) for data on precipitation and Robert Tesh for review of the manuscript.

This study was supported by the Fundação Nacional de Saúde (FNS)/IEC.

Dr. Travassos da Rosa was chief of the Arbovirus Department at Instituto Evandro Chagas and Director of the WHO Collaborating Center of Arbovirus there from 1979 to 1998. Since 1998, she has been a visiting scientist in the Department of Pathology, Center for Tropical Diseases, University of Texas Medical Branch in Galveston. Her research interests focus on the serology and taxonomy of arboviruses and hantaviruses.

### References

1. Pinheiro FP, Nelson M. Re-emergence of dengue and dengue haemorrhagic fever in the Americas. Dengue Bulletin, World Health Organization (New Delhi) 1997:21:16-24.
2. Shope RE. The use of a microhemagglutination-inhibition test to follow antibody response after arthropod-borne virus infection in a community of forest animals. Anais de Microbiologia (Rio de Janeiro) 1963;11(parte A):167-71.
3. Tesh RB. A method for the isolation and identification of dengue viruses, using mosquito cell cultures. Am J Trop Med Hyg 1979:28:1053-9.
4. Kuno G, Gomez I, Gubler DJ. Detecting artificial antidengue IgM immune complexes using an enzyme-linked immunosorbent assay. Am J Trop Med Hyg 1987:36:153-9.
5. Osanai CH, Travassos da Rosa APA, Tang AT, Amaral RS, Passos AC, Tauil PL. Surto de dengue em Boa Vista, Roraima. Rev Inst Med Trop Sao Paulo 1983:25:53-4.
6. Vasconcelos PFC, Travassos da Rosa ES, Travassos da Rosa JFS Freitas RB, Dégallier N, Rodrigues SG, et al. Epidemia de febre clássica de dengue causada pelo sorotipo 2 em Araguaína, Tocantins, Brasil. Rev Inst Med Trop Sao Paulo 1993:35:141-8.
7. World Health Organization. Dengue haemorrhagic fever: diagnosis, treatment and control. Geneva: The Organization; 1986. p. 58+.
8. World Health Organization. Dengue haemorrhagic fever: diagnosis, treatment and control. 2nd ed. Geneva: The Organization; 1997. p. 84+.
9. Gubler DJ. Dengue and dengue hemorrhagic fever. Clin Microbiol Rev 1998;11:480-96.

10. Beaty BJ, Calisher CH, Shope RE. Arboviruses. In: Schmidt NJ, Emmons E, editors. Diagnostic procedures for viral, rickettsial and chlamydial infections, 6th ed. Washington: American Public Health Association; 1989: p. 797-855.
11. Causey OR, Theiler M. Virus antibody survey on sera of residents of the Amazon Valley in Brazil. Am J Trop Med Hyg 1958:7:36-41.
12. Gubler DJ. Vigilancia activa del dengue y de la dengue hemorragica del dengue. Boletin de la Oficina Sanitaria Panamericana 1989:107:22-30.
13. Fundação Nacional de Saúde. Casos de dengue e febre amarela no Brasil. Brasília: Ministério da Saúde; 1999.
14. Gubler DJ. Epidemic dengue/dengue haemorrhagic fever: a global public health problem in the 21st Century. Dengue Bulletin, World Health Organization (New Delhi) 1997:21:1-15.
